# Visual and Refractive Outcomes of a Toric Presbyopia-Correcting Intraocular Lens

**DOI:** 10.1155/2016/7458210

**Published:** 2016-01-13

**Authors:** Alice T. Epitropoulos

**Affiliations:** ^1^The Eye Center of Columbus, 262 Neil Avenue, Columbus, OH 43215, USA; ^2^The Ohio State University, Columbus, OH, USA

## Abstract

*Purpose*. To evaluate outcomes in astigmatic patients implanted with the Trulign (Bausch + Lomb) toric presbyopia-correcting intraocular lens (IOL) during cataract surgery in a clinical practice setting.* Methods*. Retrospective study in 40 eyes (31 patients) that underwent cataract extraction and IOL implantation in a procedure using intraoperative wavefront aberrometry guidance (ORA system). Endpoints included uncorrected visual acuity (VA), reduction in refractive cylinder, accuracy to target, axis orientation, and safety.* Results*. At postoperative month 1, refractive cylinder was ≤0.50 D in 97.5% of eyes (≤1.00 D in 100%), uncorrected distance VA was 20/25 or better in 95%, uncorrected intermediate VA was 20/25 or better in 95%, and uncorrected near VA was 20/40 (J3 equivalent) or better in 92.5%. Manifest refraction spherical equivalent was within 1.00 D of target in 95% of eyes and within 0.50 D in 82.5%. Lens rotation was <5° and best-corrected VA was 20/25 or better in all eyes.* Conclusion.* The IOL effectively reduced refractive cylinder and provided excellent uncorrected distance and intermediate vision and functional near vision. Refractive predictability and rotational stability were exceptional. Implantation of this toric presbyopia-correcting IOL using ORA intraoperative aberrometry provides excellent refractive and visual outcomes in a standard of care setting.

## 1. Introduction

Corneal astigmatism affects a significant proportion of patients undergoing cataract surgery; studies have estimated that 22% to 25% of cataract patients have more than 1.50 D of corneal astigmatism [1, 2]. Because residual postoperative refractive astigmatism compromises visual outcomes, concurrent reduction of astigmatism is vital to achieving patient satisfaction following cataract surgery. Techniques to assist with reduction of astigmatism include limbal relaxing incisions (LRIs), astigmatic keratotomy, excimer or femtosecond laser refractive surgery, and toric intraocular lenses (IOLs) [3].

Toric IOLs are generally a predictable treatment for astigmatism [3] and their use prevents the development of irregular astigmatism that may result from corneal manipulation, as well as potential complications associated with incisions, such as exacerbation of dry eye, variable wound healing, and infection. Toric IOLs also have the advantage of potential reversibility. Several monofocal toric IOLs are available. These IOLs effectively reduce astigmatism [4, 5], but if corrected for distance VA, patients typically still rely on glasses for computer work and reading.

Premium lenses, either accommodative or multifocal, address presbyopia and provide a broader range of vision. The Trulign toric IOL (Bausch + Lomb, Bridgewater, NJ, USA) is a toric modification of the Crystalens accommodative IOL (Bausch + Lomb) with a toric optic on the posterior surface. The IOL was designed to reduce postoperative refractive cylinder and provide improved distance, intermediate, and near vision. The aspheric optic of the parent IOL provides excellent image sharpness [6] and depth of focus [7]. On the basis of favorable refractive and visual outcomes in a FDA registration trial [8], in 2013, the Trulign toric IOL became the first premium presbyopia-correcting toric IOL available for use in the United States.

The objective of this study was to evaluate the efficacy and safety of the Trulign toric IOL in astigmatic cataract patients implanted with the IOL in a standard of care, clinical practice setting.

## 2. Materials and Methods

### 2.1. Patients

This retrospective, noncomparative study involved patients who underwent phacoemulsification and implantation of the Trulign toric IOL in one or both eyes at The Eye Center of Columbus (Columbus, OH, USA) between August 2013 and October 2014. The study was approved by the Mount Carmel Institutional Review Board and was conducted in accordance with the tenets of the Declaration of Helsinki. All patients provided informed consent before undergoing surgery.

The inclusion criteria included patients who underwent phacoemulsification and implantation of the Trulign toric IOL; use of the intraoperative wavefront aberrometer (ORA System; WaveTec Vision, Alcon, Aliso Viejo, CA, USA) was required. Eyes that underwent procedures concomitant with the phacoemulsification and lens implantation were excluded.

### 2.2. Intraocular Lens

The Trulign toric IOL (model BL1UT, Bausch + Lomb) is a silicone multipiece IOL that is a toric modification of the Crystalens accommodative IOL; the only differences are that the posterior surface of the optic is toric, and the anterior surface of the optic has two marks that indicate the flat meridian of the lens and aid in alignment. The plate haptics are hinged adjacent to the optic and have small polyimide loop haptics. The overall diameter of the IOL is 11.5 mm and the optic diameter is 5.0 mm. The toric presbyopia-correcting IOL is available in spherical equivalent (SE) powers ranging from +10.00 to +33.00 D in 0.50 D increments, with cylindrical powers of 1.25 D, 2.00 D, and 2.75 D at the lens plane (estimated cylindrical powers of 0.83 D, 1.33 D, and 1.83 D, resp., at the corneal plane). The recommended starting A-constant is 119.1.

### 2.3. Preoperative Assessment

A comprehensive eye examination conducted preoperatively included a detailed history, slit-lamp biomicroscopy, and ophthalmoscopy. Patients were administered the validated Standard Patient Evaluation of Eye Dryness (SPEED) questionnaire [9] to screen for the presence of ocular surface disease, and patients with ocular surface disease began treatment tailored to the type and severity of dry eye. Dry eye treatments used most commonly were fish oil (reesterified omega-3 fatty acids), thermal pulsation, topical corticosteroid, topical cyclosporine, and punctal occlusion. Treatment was continued until the ocular surface was healthy enough to generate accurate measurements (on average, 4–6 weeks).

Keratometry, topography, axial length, and anterior chamber depth measurements were taken to determine the power of IOL to be implanted. If topography and keratometry measurements were not consistent, the surgery was delayed. The Trulign calculator [10] estimated the toric IOL cylinder power and lens axis orientation needed to best correct for the predicted corneal cylinder, based on keratometry, the incision location, and a predicted magnitude of surgically induced astigmatism (SIA) of 0.30 diopters.

### 2.4. Surgical Technique

All surgeries were performed by one surgeon (Alice T. Epitropoulos) under topical anesthesia using standard phacoemulsification technique. Cardinal reference marks to help with axis of lens placement were made preoperatively on the limbus at 12, 3, 6, and 9 o'clock meridians of the cornea. A Mastel marker (Mastel Precision Surgical Instruments, Inc., Rapid City, SD) was used to mark the steep axis of astigmatism as determined by preoperative testing. A 2.8 mm clear corneal, three-plane incision was created temporally at 10° in left eyes and 190° in right eyes. A cohesive ophthalmic viscoelastic device (OVD) (ProVisc; Alcon, Fort Worth, TX, USA) was injected into the anterior chamber, and a round anterior continuous curvilinear capsulorhexis of 5.2–5.5 mm was created manually around the visual axis with Utrata forceps. Coaxial phacoemulsification and extraction of the cataract was performed using an Alcon Infiniti phacoemulsification unit (Alcon, Fort Worth, TX, USA). Meticulous cortical cleanup was followed by polishing of the anterior and posterior capsule using the Whitman/Shephard Capsule Polisher (Bausch + Lomb). Intraoperative wavefront aberrometry was used in all surgeries to corroborate the preoperative assessments of sphere and cylinder in the aphakic eye, and also to confirm correct alignment of the axis of the IOL. When the recommendation of ORA differed from that of preoperative measurements and the Trulign calculator, we decided which recommendation to follow on a case-by-case basis. Most often when there was a discrepancy, we used a compromise halfway between the differing recommendations.

The presbyopia-correcting toric IOL was placed in the capsular bag, spun to ensure that the haptics were at the equator, and then rotated to obtain correct alignment relative to the markings on the cornea and the steep axis of astigmatism. A posttoric measurement was taken using the intraoperative aberrometer to evaluate and aid in the final placement of the toric IOL. After the toric lens placement was confirmed and finalized, all OVD was removed from the eye including behind the IOL. We confirmed that the Trulign IOL was vaulted posteriorly in the capsule, and the toric alignment was once again verified. The wound was tested for integrity and if not watertight, the incision was closed with either a suture or sealant (ReSure; Ocular Therapeutix, Inc., Bedford, MA).

### 2.5. Postoperative Assessment

All patients were followed for a minimum of 1 month after surgery. Preoperative and postoperative month 1 data were collected from patient charts for analysis. When available, data also were collected from follow-up at 3–13 months after surgery.

The main outcome measures included corneal and manifest refractive cylinder, refractive predictability, uncorrected distance visual acuity (UDVA), uncorrected intermediate visual acuity (UIVA) measured at 70–80 cm, uncorrected near visual acuity (UNVA) measured at 40 cm, IOL rotational stability, and safety parameters (adverse events, surgical complications, and best-corrected visual acuity (BCVA)). As part of the safety evaluation, patients were evaluated for visual disturbances, and all patients were asked specifically if they had any problems with night vision.

Rotational stability of the lens was evaluated at a slit-lamp (Haag-Streit, Mason, OH) that has degree marks labeled on the beam, which allows measurement of the toric IOL axis when the slit beam is aligned with the toric IOL axis markings in a dilated eye [11].

## 3. Results

This case series included 40 eyes in 31 patients. The mean (± standard deviation, SD) age of the patients was 71.2 ± 5.2 years (range: 57–80). Thirteen patients (41.9%) were male and 18 (58.1%) female. Five eyes were post-LASIK, 2 eyes had a history of epiretinal membrane, 2 eyes had mild irregular astigmatism that was not felt to be associated with substantial visual disturbance, and 1 eye had a history of macular pucker and trans-pars plana vitrectomy. The surgeon considered all eyes to have potential for 20/32 or better BCVA. Mean preoperative K cylinder was 1.77 D (range from 0.63 to 2.77 D). Mean preoperative manifest refraction spherical equivalent (MRSE) was −1.01 D (range from −6.25 to 3.13 D). Mean axial length was 24.4 mm (range from 22 to 27 mm). All eyes were implanted with the toric presbyopia-correcting IOL during routine cataract surgery.

The mean target MRSE for operated eyes was −0.30 D (range, plano to −0.64 D). The target MRSE was between plano and −0.30 in 55% of eyes and between plano and −0.50 D in 87.5% of eyes. Intraoperative aberrometry results affected the selection of the spherical or cylindrical power of IOL used, or the alignment of the lens, in approximately half of the eyes. There were no intraoperative surgical complications.

### 3.1. Refractive Outcomes

**Figure 1:**
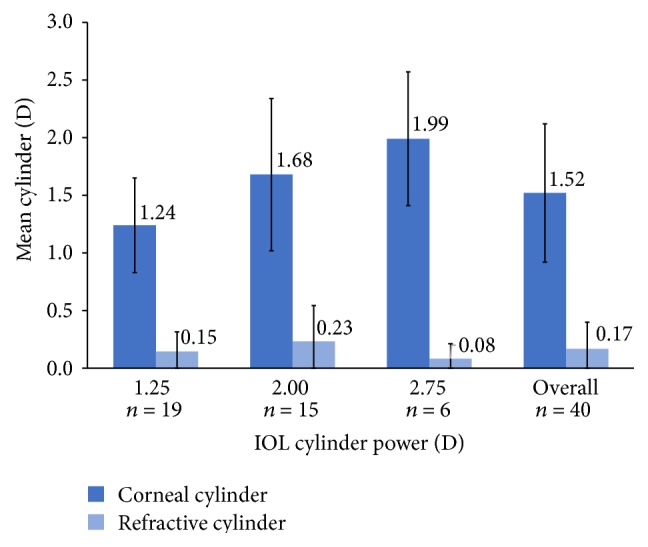
Mean corneal and refractive cylinder at postoperative month 1.

**Figure 2:**
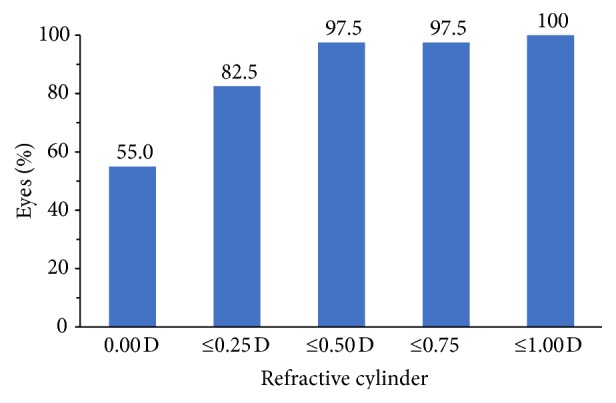
Frequency distribution of residual refractive cylinder at postoperative month 1.

At postoperative month 1, mean MRSE was −0.12 D. Mean (± SD) corneal cylinder was 1.52 ± 0.60 D, and mean refractive cylinder was 0.17 ± 0.23 D. At each lens cylinder power (1.25, 2.00, and 2.75 D), the toric lens effectively neutralized the effects of corneal cylinder on postoperative refraction (Figure 1). Refractive cylinder at postoperative month 1 was ≤0.5 D in 97.5% (39/40) of eyes and ≤1.00 D in all 40 eyes (Figure 2).

**Figure 3:**
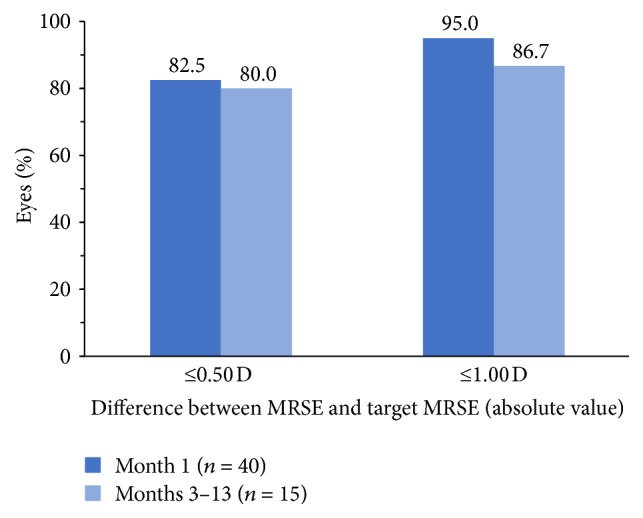
Refractive predictability of the IOL (absolute difference between MRSE and target MRSE). IOL: intraocular lens; MRSE: manifest refraction spherical equivalent.

The IOL demonstrated good refractive predictability (Figure 3). At postoperative month 1, MRSE was within 0.50 D of the target MRSE in 82.5% (33/40) of eyes and within 1.00 D of the target MRSE in 95% (38/40) of eyes. In the 15 eyes with longer-term follow-up, MRSE was within 0.50 D of the target MRSE in 80% (12/15) of eyes and within 1.00 D of the target MRSE in 86.7% (13/15) of eyes at 3–13 months after surgery.

### 3.2. Visual Outcomes

**Figure 4:**
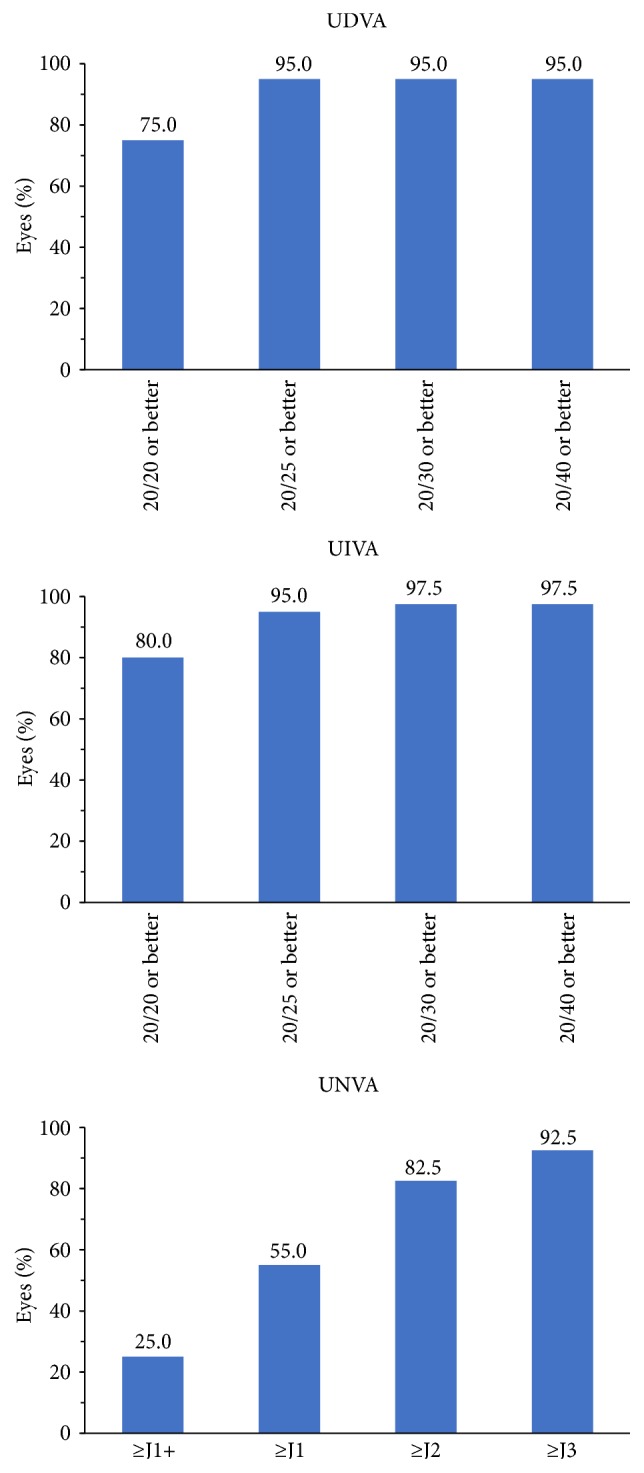
Uncorrected distance, intermediate, and near visual acuity (UDVA, UIVA, and UNVA) at postoperative month 1.

At postoperative month 1, UDVA was 20/20 or better in 75% (30/40) of eyes and 20/25 or better in 95% (38/40) of eyes (Figure 4). UDVA in the remaining 2 eyes was worse than 20/40. One of these eyes had a MRSE of −1.50 D, and UDVA was 20/70; however, the patient enjoyed her excellent uncorrected reading vision. Her UIVA was 20/16, and UNVA was 20/20. The other patient had unexpected hyperopia (MRSE was +1.38 D). Both patients were offered an IOL exchange but were satisfied with using glasses and declined the lens exchange.

Intermediate visual acuity results were exceptional. At postoperative month 1, UIVA was 20/20 or better in 80% (32/40) of eyes, 20/25 or better in 95% (38/40) of eyes, and 20/40 or better in 97.5% (39/40) of eyes (Figure 4). Near visual acuity results were also favorable. Overall, 92.5% (37/40) of all eyes had UNVA of J3 or better at postoperative month 1 (Figure 4), and UNVA in the remaining 3 eyes was J5.

Quality of vision was excellent in all eyes. There were no complaints of glare, halos, problems with night vision, or other visual disturbances.

### 3.3. Rotational Stability

At the postoperative month 1 evaluation, lens rotation was <5° in all eyes.

### 3.4. Safety

There were no unexpected adverse events related to the procedure or lens. One eye with a history of epiretinal membrane developed early capsular fibrosis that required Nd:YAG laser capsulotomy. Another eye with a history of irregular astigmatism had residual refractive cylinder noted at postoperative day 7. The lens was in the original position where we placed it (it had not rotated). Using the Berdahl & Hardten Toric IOL Calculator [12], we determined that the lens needed to be rotated from 95 to 120 degrees to better neutralize the cylinder. We performed a secondary surgical intervention rotating the lens 25°. MRSE was plano and UDVA was 20/20 in the eye at postoperative month 1.

BCVA was 20/20 or better in 92.5% (37/40) of eyes and 20/25 or better in all 40 eyes at postoperative month 1.

## 4. Discussion

The presbyopia-correcting, toric IOL demonstrated excellent refractive predictability in this study. The IOL effectively neutralized the postoperative corneal cylinder, with mean postoperative refractive cylinder reduced to near zero. Visual outcomes were favorable. The targeted MRSE of plano to −0.64 D helped to achieve acceptable near vision without compromising distance vision. Uncorrected distance and intermediate visual acuity and quality of vision were excellent.

The results of this study are consistent with the favorable safety and efficacy outcomes demonstrated in the FDA registration trial of the lens [8]. The inclusion and exclusion criteria for this study were chosen to have a study population somewhat similar to the study population in the FDA trial yet still reflect real-world clinical practice. The mean age of patients in this study was similar to that in the registration trial, but the ophthalmic histories of patients in this study were more complicated, reflecting real-world clinical practice. Altogether, 25% of eyes in this study would have been excluded from the registration trial because of irregular corneal astigmatism (the toric IOL is recommended for use in patients with regular astigmatism only), previous refractive surgery, macular pathology, and previous vitrectomy. Although eyes with these characteristics are generally not considered to be candidates for a premium lens, in our experience, the toric lens can be used successfully in such eyes when patients are appropriately counseled and have realistic expectations.

Despite the inclusion of eyes that would not have been eligible for a multifocal lens, the refractive and visual outcomes in this study were outstanding. The mean residual refractive cylinder at postoperative month 1 was 0.17 D in this series, compared with a postoperative month 4–6 mean residual cylinder value of 0.43 D (all toric IOL powers) in the registration trial [13]. The residual refractive cylinder was ≤0.50 D for 97.5% of eyes in this study, and 55% of eyes had complete resolution of astigmatism, compared with 70.9% of eyes with residual refractive cylinder ≤0.50 D and 34.3% of eyes with complete resolution of astigmatism in the registration trial [8]. The refractive predictability of the lens was outstanding in both studies. Postoperative MRSE was within 0.50 D of the target in 82.5% of eyes in this study and 73.7% of eyes in the FDA registration trial [13].

In this case series, 95% of eyes achieved 20/25 or better UDVA and 95% achieved 20/25 or better UIVA, compared with 72.4% and 86.6%, respectively, in the registration trial [8]. Quality outcomes were particularly evident in assessments of near vision. In this case series, 55% of eyes achieved UNVA of ≥J1 (20/25 Snellen equivalent) and 92.5% achieved UNVA of ≥J3 (20/40 Snellen equivalent). In the registration trial, 17.9% of eyes achieved UNVA of 20/25 (J1) or better, and 70.1% achieved UNVA of 20/40 (J3) or better [8].

Outcomes in this case series met or surpassed those in the registration trial in all categories, and UNVA was improved without compromising distance vision. To some degree, this is expected because of the greater variability of target MRSE used. The outstanding UDVA, UIVA, and UNVA outcomes in this case series might also be explained in part by the emphasis we placed on obtaining reliable biometry and topography measurements. Ocular surface disease was treated preoperatively to maximize the accuracy of the preoperative measurements, and, in a few patients, surgery was delayed due to inconsistent measurements. Also, intraoperative wavefront aberrometry was used in all surgeries for corroboration of preoperative measurements. When there was a discrepancy between preoperative and ORA calculations, the surgeon determined which calculations to use, and use of the ORA calculations did not necessarily always result in better outcomes. Nonetheless, use of intraoperative wavefront aberrometry influenced the choice of lens power or axis placement in approximately half of the cases and may have improved our outcomes overall.

Lens positioning and rotational stability is crucial because even small errors in positioning or rotation have the potential to affect the uncorrected visual acuity. The rotational stability of the toric lens was excellent in this case series, as well as in the registration trial. The FDA trial utilized photographs to evaluate for rotational stability. In the FDA registration trial, 96.1% of the implants rotated less than 5 degrees from implantation to 4–6 months postoperatively [8]. The polyimide loop haptics allow for excellent rotational stability with this IOL platform. Meticulous cortical cleanup is critical in preventing capsular fibrosis. Rigorous polishing of the anterior and posterior capsule removes the stimulus for the anterior capsule to fibrose and contract, minimizing the potential for the lens to move or tilt. We recommend early Nd:YAG laser capsulotomy for capsular fibrosis that develops, because asymmetric fibrosis can shift the lens in an asymmetric manner.

The toric IOL, built on the accommodative Crystalens IOL platform, provided excellent visual quality in this case series. The aspheric optics of the parent lens have been associated with excellent quality of vision, including better contrast sensitivity and fewer problems with glare and halos, compared with multifocal lenses [14]. Because multifocal IOLs are often associated with loss of contrast sensitivity [15], they may not perform as well at night and should not be implanted in patients with macular pathology [3]. Unfortunately, it is difficult to predict whether a patient will develop macular pathology. An accommodative IOL can be used for patients with macular pathology, and the toric IOL can be used in patients with preoperative corneal astigmatism who desire an excellent range of vision. It is also an ideal lens for cataract patients with preoperative corneal astigmatism and a monofocal lens in the contralateral eye, who desire a broader range of vision.

A limitation of this study is the lack of a control group, which is a common limitation in a retrospective case series study design. Multifocal toric IOLs are not yet available for use in the United States, but future prospective studies should evaluate the Trulign toric IOL compared with a multifocal toric IOL in patients with preoperative corneal astigmatism who desire a range in vision and would accept either the Trulign toric or a multifocal toric IOL.

## 5. Conclusions

The availability of a premium presbyopia-correcting IOL that offers toric correction is an important advancement in patient care. In this case series, the novel toric IOL provided excellent UDVA and UIVA and functional UNVA. The lens effectively and predictably reduced refractive astigmatism and demonstrated excellent rotational stability, and no patient had visual disturbances. Use of this presbyopia-correcting toric IOL can provide excellent refractive and visual outcomes in a standard of care, clinical practice setting. This toric IOL is an excellent option for astigmatic patients undergoing cataract surgery who desire a wide range of vision along with quality night vision.
